# Efficacy of Dietary and Supplementation Interventions for Individuals with Type 2 Diabetes

**DOI:** 10.3390/nu13072378

**Published:** 2021-07-12

**Authors:** Jessica Lewgood, Barbara Oliveira, Marie Korzepa, Scott C. Forbes, Jonathan P. Little, Leigh Breen, Robert Bailie, Darren G. Candow

**Affiliations:** 1 Faculty of Kinesiology and Health Studies, University of Regina, Regina, SK S4S0A2, Canada; jessicalewgood@gmail.com (J.L.); Robert.Bailie@uregina.ca (R.B.); 2 Okanagan Campus, School of Health and Exercise Sciences, University of British Columbia, Kelowna, BC V1V1V7, Canada; barbara.oliveira@ubc.ca (B.O.); jonathan.little@ubc.ca (J.P.L.); 3 School of Sport, Exercise and Rehabilitation Sciences, University of Birmingham, Edgbaston, Birmingham B15 2TT, UK; MXK098@student.bham.ac.uk (M.K.); L.Breen@bham.ac.uk (L.B.); 4 Department of Physical Education Studies, Faculty of Education, Brandon University, Brandon, MB R7A6A9, Canada; ForbesS@BrandonU.CA

**Keywords:** insulin, glucose, metabolism

## Abstract

The prevalence of Type 2 diabetes (T2D) is increasing, which creates a large economic burden. Diet is a critical factor in the treatment and management of T2D; however, there are a large number of dietary approaches and a general lack of consensus regarding the efficacy of each. Therefore, the purpose of this narrative review is twofold: (1) to critically evaluate the effects of various dietary strategies on diabetes management and treatment, such as Mediterranean diet, plant-based diet, low-calorie and very low-calorie diets, intermittent fasting, low-carbohydrate and very low-carbohydrate diets, and low glycemic diets and (2) to examine several purported supplements, such as protein, branched-chain amino acids, creatine, and vitamin D to improve glucose control and body composition. This review can serve as a resource for those wanting to evaluate the evidence supporting the various dietary strategies and supplements that may help manage T2D.

## 1. Introduction

Diabetes encompasses a spectrum of disorders represented most prominently by an inability to properly regulate blood glucose due to an impaired response to utilize insulin, the hormone responsible for transporting glucose from the blood into cells [1]. It is estimated that >170 million people will have diabetes by the year 2030 [2], with the highest prevalence occurring in older adults (<65 years of age) [3]. The vast majority of these individuals have type 2 diabetes (T2D) [4]. T2D is characterized by the impairment of insulin secreting β-cells in the pancreas as well as a blunted response to insulin [4]. Post-prandial hyperglycemia is a primary characteristic of T2D in older adults. While multifactorial, the age-related reduction in muscle mass/quality and muscle function may be contributing factors for developing T2D in older adults [5]. While some forms of diabetes are inherently genetic, T2D can be caused by factors associated with nutrition and physical inactivity [6]. It is well established that modifications to habitual dietary practices play an important role in the treatment and management of T2D; however, there are a large number of dietary approaches and general lack of consensus regarding the efficacy of each strategy, especially in older adults. Furthermore, conducting nutritional research is challenging and, when examining complete dietary changes, it is often difficult to identify cause and effect. Therefore, the purpose of this narrative review is twofold: (1) to critically evaluate the effects of various dietary strategies (i.e., Mediterranean; plant-based; low- and very-low calorie; intermittent fasting; low- and very-low carbohydrate, low-glycemic) on the management and treatment of diabetes, and (2) examine the efficacy and possibility of select dietary supplements (i.e., proteins, branched-chain amino acids, creatine, vitamin D) for treating T2D.

## 2. Potential Dietary Strategies for Type 2 Diabetes

### 2.1. Mediterranean Diet

Potential beneficial effects from the Mediterranean diet (MedDiet) in disease-state populations, specifically those with cardiovascular disease (CVD), was first hypothesized by Keys et al. in 1980 [7]. The MedDiet is an expression of different food cultures present in the Mediterranean region, with diverse food consumption and production patterns, in continuous evolution, representing the particular historical and environmental mosaic that is the Mediterranean [8]. UNESCO inscribed the Mediterranean diet in 2010 and again in 2013 on the list of the Intangible Cultural Heritage of Humanity, amplifying the significance of this regimen as a lifestyle intervention [9]. The diet is characterized by abundant plant foods (vegetables, legumes, nuts), cereals/whole grains (mainly unrefined), fresh fruit, olive oil as the principal source of fat, low to moderate intake of dairy products (principally cheese and yogurt), and fish and poultry consumed in low to moderate amounts, some eggs, red meat consumed in low amounts, and wine consumed in low to moderate amounts, normally with meals. This diet is low in saturated fat (<or = 7–8% of energy), with total fat ranging from >25% to <35% of energy throughout the region [10,11].

Adherence to a MedDiet has been associated with a reduction in mortality [12], increased longevity [13] and prevention and management of T2D and Metabolic Syndrome [14]. The efficacy of a MedDiet in the management and prevention of T2D is now one of the most widely investigated dietary patterns amongst researchers in this area. A number of review articles have summarized the existing evidence regarding the efficacy of the MedDiet for improving cardiometabolic health in T2D and prediabetes [15,16,17,18]. Both observational and intervention studies tend to support benefits, particularly for people with T2D.

Associative evidence: A meta-analysis from 2018 concluded that the MedDiet appeared to be the most effective and efficacious dietary approach to improve glycemic control in people with T2D [19]. In addition, Esposito et al. [16] associated the MedDiet with lower HbA1c levels and improved cardiovascular risk factors, as compared with control diets, making it a dietary pattern suitable for the overall management of T2D [16]. A role for the MedDiet in the prevention of CVD outcomes has been extensively studied in several randomized controlled trials (RCTs) and prospective cohort studies, but recently Becerra-Tomás et al. [20] demonstrated its beneficial role in reducing risk, incidence and mortality from various CVD outcomes, specifically in individuals with diabetes [20]. Subsequently, there is associative evidence that the MedDiet provides benefits to cardiometabolic health and reduced mortality in people with T2D.

Intervention studies: Greco et al. [21] found that a 4-month moderate caloric restriction diet (1400–1600 kcal/day) in obese individuals that followed a MedDiet significantly improved body weight and body mass index (BMI), insulin, and insulin sensitivity (HOMA-IR). Vitale et al. [22] analyzed 2568 participants with T2D and concluded that a MedDiet was associated with favorable cardiovascular risk factors profile, better glucose control and lower BMI. The MÉDITA trial [23], a single-center, randomized, controlled trial designed to investigate whether a MedDiet reduces the need for drug therapy in individuals with T2D, concluded that the MedDiet ameliorates the inflammatory milieu of T2D and Homeostatic Model Assessment (HOMA). The Cordioprev study [24] randomized patients with T2D to a low-fat diet (*n* = 130) or a MedDiet (*n* = 111) [25]. Results showed that the MedDiet improved post-prandial lipidemia and provided evidence of improvements in insulin resistance. The PREDIMED study [26] is the largest dietary intervention trial to assess the effects of the MedDiet on cardiovascular disease prevention. Many papers have been published from this study with the main paper concluding that an unrestricted-calorie, high-vegetable-fat MedDiet was associated with less gain in central adiposity and improved fasting glucose in participants with T2D along with decreased insulin resistance in those without diabetes.

Other studies such as The PREDIMED-Plus trial [27,28] and the Dietary Intervention Randomized Controlled Trial group (DIRECT) in Israel [29] used a MedDiet as the dietary intervention, but in an energy restricted context. The PREDIMED-Plus trial was a 6-year parallel-group, multicenter RCT involving 6874 participants recruited in 23 Spanish recruiting centers. The trial revealed improvements in glycemic control, insulin sensitivity, and dyslipidemia in individuals with or at risk for diabetes. In the Israeli study, 322 participants with moderate obesity were randomized for 2 years to one of three diet groups: low-fat, MedDiet or low-carbohydrate. Differential effects were observed in the sub-group of patients with T2D at 24 months: participants randomized to the MedDiet, which had the highest intake of dietary fibers and unsaturated to saturated fat ratio, achieved significantly greater improvements in fasting plasma glucose and insulin levels. Furthermore, special features (via dietary polyphenols and dietary fibers provided by key components of the MedDiet such as olive oil, nuts, red wine, legumes, fruits, and vegetables) have apparent additional favorable effects for patients with T2D such as reduced fasting plasma glucose and lower HbA1c levels [30].

Collectively, research findings indicate that MedDiet has positive effects on several outcome measures associated with metabolic health [31], is a viable dietary strategy for the management of T2D [16] and may reduce the risk of pre-mature mortality for individuals with T2D [32]. Moving forward, research studies should determine which components of the MedDiet generate specific health benefits. For example, are the metabolic and health benefits related to specific components of a MedDiet (e.g., olive oil, nuts)? Are these purported benefits the result of a shift from a highly processed “Western”-style diet to an unprocessed MedDiet? Or could the benefits be the results of better adherence to the MedDiet?

### 2.2. Plant-Based Diets

Plant-based diets aim to maximize consumption of nutrient-dense plant foods while minimizing processed foods, oils, and animal foods (including dairy products and eggs). In general, these diets emphasize vegetables, fruits, beans, peas, lentils, soybeans, seeds, and nuts. There is evidence that a broadly defined plant-based diet can have significant health benefits [33]. It should be noted that the term “plant-based” is sometimes used interchangeably with vegetarian or vegan. Among these approaches, macronutrient intakes are commonly distributed as 35–40% fat, 16–17% protein and 40–50% carbohydrates, with a high ingestion of fibers (15–20 g/day) [34]. As with all types of diets, vegetarian or vegan diets adopted for ethical, religious, or other reasons may or may not be healthy [35].

Plant-based diets have received increasing interest for their use in the management of T2D. Diabetes Canada endorses plant-based diets for the management of T2D because of their potential to have favorable effects on body weight, HbA1c, LDL-cholesterol, total cholesterol and non-HDL-cholesterol levels [36]. However, it is important to note that refined grains, starches, and sugars can also be characterized as plant-based, although they are independently associated with a higher risk for developing T2D [37]. Hence, the effects of plant-based diets on the prevention and treatment of T2D are not universal and requires further research [38].

Associative evidence: Vegetarian diets are often associated with weight loss, as is the case with most calorie deficit diets [34,39,40], making it difficult to assume that any improvement in glycemic control is related to the plant-based diet per se. A meta-analysis comparing different dietary approaches, including the vegetarian diet, did not conclude its superiority over diets such as MedDiet, low-carbohydrate or Paleolithic in weight loss or various glycemic parameters [19]. However, diets with higher ingestion of plant-based foods and reduced intake of animal-based foods have been associated with lower risk for cardiovascular morbidity and mortality [41,42,43]. The American Heart Association, American Diabetes Association (ADA) and the European Association for the Study of Diabetes (EASD) recognize the benefits of eating a plant-based diet as long as food choices are nutritious [44,45]. A recent systematic review suggested that a shift to a plant-based diet may lead to favorable effects on glycemic control in individuals diagnosed with T2D [46]. Similar findings were obtained by Qian et al. supporting the health benefits of increased plant-based food consumption in lowering the risk of T2D and other cardiometabolic diseases [38].

Intervention studies: Body weight, HbA1c and cardiovascular benefits of plant-based diets became popularized in 2006 after the study by Barnard et al. [39]. More recently, the BROAD study [47] compared standard medical care (control) to standard medical care plus a diet change program (intervention) of a low-fat, plant-based diet. The intervention led to significant and sustained reductions in body mass index (BMI: −3.9 kg/m^2^), body weight and cholesterol (−0.45 mmol/L) compared with the control group. Several other trials in participants with T2D also report weight loss and improvements in markers of insulin resistance following plant-based dietary interventions [34,40,41].

Plant-based diets improve weight status and increase dietary fiber but limit heme iron and vitamin B12 ingestion [48]. Thus, assistance in meal planning to minimize risk of nutrient deficiencies, maximize glycemic control, and address individual dietary preferences is recommended. Overall, the impact of plant-based diets (broadly defined) on the prevention and management of T2D and its complications show some promise, but still requires further investigation.

### 2.3. Low-Calorie Diets

Low-calorie diets (LCD), also referred to as low-energy diets, hypocaloric diets, or calorie restriction diets, have a scientific record for treating obesity-related conditions dating back to the late 1920s, where the 300 to 400 kcal/day diet of Strang et al. produced average weight losses of 9.9 kg in 8 weeks in a large series of patients [49].

Calorie restriction is the only known strategy to robustly improve health and lifespan in most, if not all, living organisms [50]. It leads to numerous changes in animal models, including alterations in body composition, energy expenditure, oxidative stress/damage, cardiovascular disease, insulin sensitivity, neuroendocrine function, and gene expression [51]. Studies involving fasting have progressed to different clinical procedures, including dietary protein or protein and carbohydrates with fasting, to preserve lean body mass [52]. This strategy led to recent definitions of very low-calorie diets or calorie restriction diets as dietary regimens low in calories without undernutrition [53].

LCDs are defined as providing 1200–1500 kcal/day whereas very low-calorie diets (VLCDs) contain energy levels <800 kcal/day with daily allowances of all essential nutritional requirements [54]. VLCDs can be designed to replace the whole diet using products (e.g., meal replacement shakes) and can be defined as a total diet replacement. In comparison, the LCD can either be a total diet replacement or the formula products can be incorporated into modified conventional meals as a partial diet replacement [55]. Macronutrient distribution varies among different approaches from very low-fat diets (about 21% protein, 8% fat and 71% carbohydrate) to low-carbohydrate diets (about 21% protein, 50% fat and 29% carbohydrate) [56].

One of the essential health outcomes for the prevention of multiple chronic diseases and the promotion of healthy aging is maintaining a healthy body weight and preventing accumulation of abdominal fat. Low-calorie regimens constitute weight loss strategies for individuals with overweight or obesity [57], a common profile in people with T2D. Weight loss produces numerous benefits such as improved glucose metabolism, reduction in glycaemia and fasting insulin levels and increased insulin sensitivity, reduction in blood pressure, and improvements in lipid profile with decreased triglycerides, increased high density lipoprotein cholesterol (HDL-C) and fewer small, dense, low density lipoprotein cholesterol (LDL-C) particles. The advantages of very low-calorie diets include rapid weight loss that could act as a motivating factor, calorie restriction-associated diuresis that can alleviate fluid retention, and probably hunger suppression by mild ketosis [58].

Associative evidence: Beyond weight loss, VLCDs have been associated with reductions in blood glucose and improvements in cardiovascular risk profile in people with T2D [59,60]. Findings suggest that changes in measures of diabetes control, HbA1c or fasting plasma glucose are primarily the result of weight loss. It is generally believed that higher calorie restriction leads to greater weight loss [61]. In addition, when considering rapid and substantial weight loss (>15 kg), emerging evidence suggests that calorie-restriction of this magnitude can lead to reversal (or remission) of T2D in effects similar to that of bariatric surgery [62]. However, sustaining a very-low calorie diet is challenging, hence the use of formula diets containing 800–900 kcal per day as meal replacements during an initial rapid weight loss phase [63].

There is still limited evidence that a VLCD can maintain weight loss over the long term. For example, meta-analysis results showed that VLCD resulted in greater weight reduction (compared to LCD) in the short-term. However, after 1 year, weight loss did not differ between dietary strategies [64].

A recent retrospective study designed to evaluate the safety, efficacy and durability of a 12 month very low-calorie ketogenic diet (VLCKD) on body weight and glycemic control in a selected group of patients with T2D and obesity showed a rapid and significant improvement in metabolic parameters, anthropometric measures and quality of life. The patients who followed a VLCKD showed a higher adherence to the prescribed nutritional regimen, compared to patients in the LCD group, probably due to the rapid weight loss and greater feeling of satiety [65]. The induction of ketosis has been associated with a greater feeling of fullness, greater tolerability, higher reduction in body fat and preservation of mean body mass [66]. Furthermore, high-ketogenic VLCDs lower glucose more than low-ketogenic VLCDs in obese T2D patients, possibly by reducing hepatic glucose output [67].

Intervention studies: Even though rapid weight loss occurs from LCDs and VLCDs [54,68], strict and life-long compliance to these dietary strategies is likely to be difficult to maintain. In order to improve adherence, VLCDs are generally used as part of a comprehensive intervention that includes an intensive weight loss phase with medical monitoring followed by a program of lifestyle modification [69,70]. The DiRECT trial, a primary care-led cluster randomized control trial, showed that a VLCD resulted in T2D remission (HbA1c < 6.5% and no glucose-lowering medications) in 46% of patients at 1-year [69] and 36% at 2 years follow up [71]. Results of the DiRECT trial highlight the concept of T2D remission which is now routinely emphasized in nutritional intervention research. In this trial, an initial phase using total meal replacement providing ~850 kcal/day through nutritional shakes, soups, and puddings was followed by a gradual food reintroduction phase with individual guidance for weight loss maintenance. Little information was provided about dietary composition during the maintenance phase at 1 and 2 years when the T2D remission data were reported, but the findings suggest a combined approach of VLCD with medical supervision and food reintroduction can produce lasting improvements in clinical T2D outcomes and possibly reverse some of the pathophysiology of the disease.

In a pilot study prior to the DiRECT trial, the Newcastle Counterpoint study achieved “reversal” of T2D with mean weight loss of 15.3 kg in 11 people with T2D within 4 years of diagnosis, using a 600 kcal/day low-energy liquid diet. The normalization of fasting plasma glucose persisted for up to 3 months after return to normal diet [72].

In addition, there is evidence that short-term caloric restriction per se improves glucose control and beta-cell function in patients with T2D and class III obesity [73]. It has also been shown that VLCDs lead to discontinued medication and optimal glycemic control with improvements in insulin sensitivity and beta cell function [74]. Efficacy of VLCDs as therapeutic intervention for weight loss and metabolic improvements in individuals with T2D is promising and consolidating. The role of VLCKDs is arising in importance and could have additional effects beyond the caloric restriction by generating a ketogenic state. However, implementation and whether such diets can be, or need to be, followed long term remain as areas of investigation for these dietary patterns in patients with T2D.

### 2.4. Intermittent Fasting

The prevention and treatment of chronic metabolic diseases through dietary approaches can also include different forms of fasting, time-restricted eating, and fasting mimicking diets with emerging evidence showing potential promise for improving clinical outcomes. These dietary practices range from time restricted eating, feeding every other day (alternate day fasting), periodic fasting (5 days eating: 2 days fasting), or undergoing a periodic cycle of diets that provide a relatively high caloric content but are designed to mimic many of the effects of fasting (Fasting Mimicking Diets) as presented in Table 1 [75].

The overall premise behind different fasting regimens is to maximize oxidation of fatty acids and ketone bodies instead of relying on glucose as a fuel source (reference required). Prolonged fasting will also result in longer periods of time where insulin levels are low, potentially assisting in weight loss and improved insulin sensitivity through reduction in hyperinsulinemia [77,78]. Weight loss could also be generated by lower caloric ingestion if there is no over-eating during eating days. A study examining intermittent fasting (5 days eating:2 days fasting) found that participants ate less than prescribed on non-fasting days (156 kcal/day) and composition of diets were around 22% of caloric intake from protein, 33% from fat and 39% from carbohydrates [79].

Intermittent fasting regimens are hypothesized to influence metabolic regulation via effects on circadian biology, weight loss, the gut microbiome, and may represent modifications to lifestyle behaviors (e.g., limit eating opportunities or overall energy intake) which may positively impact T2D [80]. Animal models show improvements in health outcome measures throughout the life span while clinical studies have focused mainly on young and middle-aged adults who are considered overweight/obese [77]. However, controversy exists regarding the efficiency of intermittent fasting as skipping breakfast (arguably a form of time restricted feeding or intermittent fasting) is associated with an elevated risk of coronary heart disease, T2D and other adverse factors [81]. Furthermore, skipping breakfast is associated with much greater glycemic spikes in response to a standardized lunch and dinner in people with T2D [82] and has been shown to disrupt certain clock genes in leukocytes [83]. Thus, the overall benefits of intermittent fasting regimens for people with T2D remain somewhat uncertain.

Associative evidence: A systematic review and meta-analysis demonstrated that intermittent fasting significantly decreased body weight when compared to a standard diet but had no greater effect on HbA1c, lipid profile or blood pressure [80,84]. These results are in accordance with another recent review [85] in which no differences were found between intermittent fasting regimens and calorie restriction. The benefits in weight reduction and improvement in glycemic control in patients with T2D do not seem to be an independent effect of IF but are similar to a standard calorie-restricted diet that results in negative energy balance.

Intervention studies: A recent review summarized five RCTs in adults with T2D and concluded that intermittent fasting and continuous energy restriction dietary practices were viable strategies for improving glycaemia and body composition measures [84]. Trepanowski et al. [86] and Sundfor et al. [87] conducted RCTs in adults with obesity and found no significant differences in weight loss, weight maintenance, or cardiovascular outcome measures when comparing alternate day fasting to daily calorie restriction. It is important to note that improvements in insulin sensitivity (reduced fasting insulin by 3.4 ± 1.6 mU/L and mean and peak insulin values by 26 ± 9 mU/L and 35 ± 13 mU/L), β-cell responsiveness (increased the insulinogenic index, a marker of β cell responsiveness, by 14 ± 7 U/mg), blood pressure, oxidative stress, and appetite have been discovered in pre-diabetic individuals who practice alternate day fasting [88]. Corley et al. examined the effects of consecutive or non-consecutive day fasting (2 days/week) for 12 weeks in individuals with T2D. Results showed that both fasting practices resulted in similar reductions in weight loss, HbA1c and fasting glucose [89]. Similar results were found in a short-term pilot study indicating that intermittent fasting may be a tolerable dietary intervention in people with T2D and could improve key outcomes including body weight (−1.395 kg), fasting glucose and postprandial variability [90]. Other randomized controlled trials show decrease in weight, but positive glycemic outcomes are not a universal finding [61,91,92,93].

An important consideration for individuals with T2D who follow any intermittent fasting protocol is safety and preventing hypoglycemia due to over-medication [57]. When adopting this regimen, people with T2D and their health care providers should be careful to adjust glucose-lowering medications, which can be challenging, as there are no universal protocols and many healthcare providers (and patients) may not be used to, or trained in, medication de-escalation.

In summary, trials investigating the impacts of IF regimens on glycemic control in patients with diabetes remain limited and results from rigorous intervention trials are scarce. It is not possible to draw any clear conclusions of benefits generated by an IF approach that surpass calorie-restriction methods.

### 2.5. Low-Carbohydrate and Very Low-Carbohydrate Diets

Although gaining popularity with the general public and the subject of significant scientific research in recent years, the use of low-carbohydrate diets in treating T2D is not new. Before the advent of insulin in 1921, carbohydrate- and calorie-restriction diets were used to manage diabetes [94,95,96]. The use of very-low carbohydrate diets regained some popularity with Dr. Atkins’ New Diet Revolution [97] and there is relatively strong evidence since then for outcomes such as weight loss and glucose management [98,99,100,101,102].

The American Diabetes Association and Diabetes Canada have recently acknowledged low- and very-low-carbohydrate diets as healthy options for weight loss and glycemic control in individuals with T2D [103]. The American Diabetes Association defines a low-carbohydrate diet as having ≤130 g of carbohydrate/day and a very low-carbohydrate diet as containing <50 g of carbohydrate/day [4]. Low-carbohydrate diets can be designed to be either normal-fat–high-protein (≥20% of caloric intake from protein) or high-fat-normal-protein. Ketogenic diets prescribe around 70% of caloric intake from fat, 15–20% protein and less than 10% carbohydrates [104]. Typically, a very low-carbohydrate diet can lead to endogenous production of ketone bodies and can be referred to as a “ketogenic” diet, although the exact level of carbohydrate intake that results in nutritional ketosis does appear to vary between individuals.

When carbohydrate content of the diet is reduced it is typical that fat and/or protein content of the diet will increase. The majority of studies tend to focus on low-carbohydrate high-fat dietary approaches, although some studies focus on low-carbohydrate high-protein diets [105,106,107].

The number of studies reporting low-carbohydrate diets as a successful therapy for weight loss, decreased insulin resistance, plasma glucose and use of glucose lowering medications are rising. Popular terms such as “diabetes remission” and “diabetes reversal” are being used to elucidate these outcomes, but the level of evidence for remission of T2D is not as strong as it is for the very low-calorie total diet replacement results from the DiRECT trial. Buse et al. [1] defined partial remission as hyperglycemia below diagnostic thresholds for diabetes for at least 1 year’s duration with no active pharmacologic therapy or ongoing procedures, and complete remission as normal glycemic measures for at least 1 year’s duration and no active pharmacologic therapy or ongoing procedures.

Associative evidence: A recent systematic review and meta-analysis concluded that patients adhering to low-carbohydrate diets for six months experience greater rates of diabetes remission, but long-term effects need monitoring and medication adjustments are required when compared with other diets commonly recommended for management of T2D [108]. These findings align with those of Silverii et al., who found that low-carbohydrate diets may produce short-term improvements in HbA1c and body weight, but are typically not maintained over the long term [109]. It is notable that the restriction of dietary carbohydrates is expected to lower blood glucose and HbA1c since consuming carbohydrates causes postprandial hyperglycemia in people with T2D [110]. The challenge among low-carbohydrate and very low-carbohydrate approaches is sustained compliance to regimens for longer period of time.

Intervention studies: Over the past 20 years, numerous studies have demonstrated the safety and efficacy of low-carbohydrate interventions for management and treatment (i.e., reducing medication use) in individuals with T2D [111]. For example, Yancy et al. showed that a low-carbohydrate diet has positive effects on body weight (−11.1 kg), waist measurement, HbA1C (−1.5%) and serum triglycerides (−42%), and fasting glucose decreased by 17% in participants with T2D [112]. Moreover, Tay et al. demonstrated that a low-carbohydrate diet had positive effects on triglycerides (−0.4 mmol/L), HDL cholesterol (+0.1 mmol/L), and glycemic control (−0.7 mmol/L in fasting glucose and −1% in HbA1c), but also lowered medication requirements and attenuated diurnal blood glucose fluctuations [113].

Short-term experimental studies have progressed to longer term RCTs which include other sub-interventions beyond only dietary interventions, which can make it difficult to isolate findings to the low-carbohydrate diet per se. One of the most well publicized trials presented one year [100] and two year [114] results from T2D participants (21–65 years) after a remote care intervention including a very low-carbohydrate diet. Following 2 years of the intervention, participants experienced improvements in HbA1c (−0.9%), fasting glucose (−1.7 mmol/L) and insulin (−11.71 mIU/L), and HOMA-IR and decreased their reliance on glucose-lowering medication classes. Furthermore, a RCT in adults with obesity and T2D showed substantial weight loss, reduced HbA1c and fasting glucose with low-calorie and low-carbohydrate diets. Only the low-carbohydrate diet resulted in significant improvements in lipid profile, blood glucose stability, and reductions in diabetes medication requirements [113].

Indeed, positive metabolic outcomes occur with weight loss as a result of different dietary approaches, and not only low-carbohydrate diets [115]. In a 12-month weight loss and diet study, there was no significant difference in weight change between a healthy low-fat diet vs. healthy low-carbohydrate diet [116]. However, very low-carbohydrate diets could advance beyond weight loss outcomes (e.g., glycemic stability, reduction in glucose-lowering medication use).

It may seem that advantage is related to the degree of carbohydrate restriction, and that very low carbohydrate diets are more effective than standard low carbohydrate diets [117]. Ketone bodies resulting from low-carbohydrate diets are used by tissues as a source of energy as an evolutionary-conserved physiological mechanism related to starvation [99]. Recent work over the last decade or so has provided accumulating evidence of the possibility of the unique therapeutic potential of ketogenic diets in many pathological conditions, such as diabetes. This supposition is supported by two recent meta-analyses of low carbohydrate diets reporting that those studies with the lowest daily carbohydrate intake found the largest reduction in HbA1c [101,118]. Participants achieving and sustaining nutritional ketosis showed improvements in blood glucose, insulin, HbA1c, weight, blood pressure, triglyceride, liver function, and inflammation and reduced dependence upon medication [114]. In addition, the ketogenic diet was most effective in reducing body weight and improving glycemic control when compared to a standard hypocaloric diet in T2D patients [119,120].

It is interesting to note that there is moderate evidence in low-carbohydrate diets as an approach that increases remission of T2D in a six-month period. Important improvements are also seen in weight loss, triglycerides, and insulin sensitivity at six months, which diminished at 12 months [108] with benefits seemingly related to the level of carbohydrate restriction, as well as compliance. Nevertheless, like other restrictive dietary patterns, long-term adherence to low-carbohydrate and very low-carbohydrate diets can be a challenge [121].

### 2.6. Low Glycemic Index Diets

Dietary recommendations have tended to emphasize the quantity rather than the quality of carbohydrate, despite the fact that carbohydrate source and type distinctly influence postprandial glycaemia. The ability of dietary carbohydrates to exacerbate postprandial plasma glucose is different and depends on their structure and fiber content. However, while dietary factors are important modifiable risk factors for T2D, the causal role of carbohydrate quality in nutrition remains controversial [122]. The glycemic index (GI) ranks the nature of carbohydrates in foods and is defined as the incremental area under the plasma glucose curve after consumption of 50 g test carbohydrate, compared with a reference food. Glycemic load (GL) is a qualitative and quantitative index computed by multiplying GI by the carbohydrate content of the food (g/100 g or 1000 kJ edible food) [123,124].

Various guidelines recommend prioritizing food with GI and GL considerations. Low GI and GL can be incorporated into healthy eating dietary advice and applied in the context of healthy diet and individualized nutritional orientation [122]. If carbohydrates are derived from low glycemic index and high-fiber foods, this has been shown to contribute to improvements in glycemic and lipid control in adults with T2D. However, even though diets with a low glycemic index may have beneficial effects on certain risk factors, the effectiveness of ad libitum consumption of low-glycemic-index diets for weight control is controversial and interventions usually follow health guidelines (45–55% carbohydrates, 10–35% protein and 20–35% fat) [125,126].

Associative evidence: Prospective cohort studies provide evidence that diets higher in glycemic index (GI) and load (GL), independently of dietary fiber, substantially elevate the risk of T2D [127]. Other studies show fiber intake as an important factor in T2D prevention. In three large prospective cohorts of males and females, there was a positive association between GI, GL, and the prevalence risk of developing T2D. Participants who consumed diets with high GI or high GL and low cereal fiber had ~40% higher risk of developing T2D compared with those whose diets were high in cereal fiber and low in GI or GL [128]. In fact, in most studies the low-GI diet was found to contain more fiber, making it difficult to separate the effects of the fiber per se from that of the low GI foods. Even though these limitations are noticed, there is still evidence that choosing low GI foods could be useful in targeting postprandial hyperglycemia [129].

As for people with impaired glucose tolerance or diabetes, a meta-analysis of 54 studies demonstrated that low GI diets reduce HbA1c, fasting blood glucose, body mass index, and blood lipids. However, the analysis failed to show a significant difference between low GI diets and control diets in lowering body weight and the low-GI diets were not superior to any individual control diet [130]. However, results have also shown that high GI diets increase triglycerides and inflammatory markers. Elevated blood glucose and insulin (with a high GI diet) can induce insulin resistance, which could lead to increased triglyceride, an inflammatory response, and a decrease in HDL cholesterol [131].

Intervention studies: RCTs have also provided evidence that lower GI diets can improve certain metabolic parameters. For example, low GI diets were effective in lowering HbA1c levels in individuals with T2D [132], whereas increases in glycemic index (with a 1-unit change) were associated with higher HbA1c levels (+0.3%) and waist circumference (+0.12 cm), but not with fasting glucose, blood lipids, or body mass index [133]. Another study with 20 participants with T2D found that consumption of a low GI diet for 30 consecutive days led to greater reductions in fat mass while a high GI diet caused an increase in fructo-samine, non-esterified fatty acids and TNF-α concentrations [134]. In a longer trial, Jenkins et al. found that a 6-month treatment with a low GI diet resulted in moderately lower HbA1c levels (−0.18%) compared with a high-cereal fiber diet in patients with T2D [135].

In patients with T2D who consumed a high-carbohydrate/high-GI diet, high-carbohydrate/low-GI diet or low-carbohydrate/high-monounsaturated-fat diet for 1 year, significant increases in disposition index, an index of beta cell function, was observed from the low-GI diet compared to the low-carbohydrate diet. No significant results were seen for high-carbohydrate/low-GI [136].

In conclusion, a low-GI diet can help manage glucose levels and parameters associated with T2D probably due to inclusion of well-balanced, minimally processed and high in fiber foods such as whole-grain, fruits and vegetables, and protein sources. While the low-GI diet can help manage glucose levels if foods are chosen wisely, in order to achieve one of the most important outcomes related to diabetes, weight loss, low-GI dietary prescriptions still have to consider reducing daily calorie ingestion.

**Figure 1:**
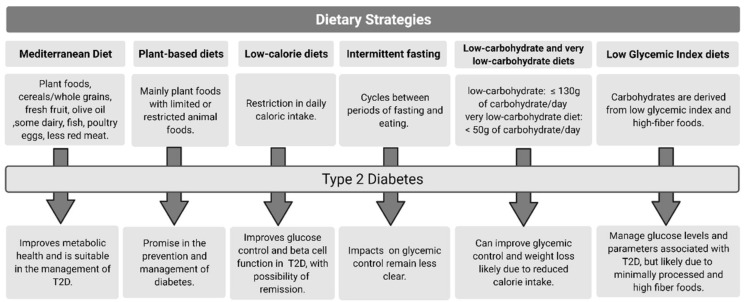
Dietary strategies and key outcomes for type 2 diabetes.

Figure 1 summarizes dietary strategies used to improve metabolic outcomes in Type 2 Diabetes.

## 3. Potential Supplements for Type 2 Diabetes

A limitation of altering complete dietary approaches is determining which nutrient(s) is associated with improvements. The purpose of this section is to outline individual dietary supplements that are purported to be effective for individuals with T2D, with a primary focus on older adults.

### 3.1. Protein Supplementation

Dietary protein has the potential to exert beneficial effects on numerous factors which underscore disease progression in older individuals with T2D. These include supporting lean muscle mass retention and accretion (a primary characteristic of sarcopenia), thereby exerting positive influence on whole-body insulin sensitivity and thermogenesis. Furthermore, dietary protein ingestion can directly impact insulin secretion and appetite regulation, both of which are crucial factors in T2D etiology.

#### 3.1.1. Dietary Protein and Lean Muscle Mass

Skeletal muscle is the largest peripheral site for glucose disposal [137]. Cross-sectional analysis of >13,000 individuals revealed 10% higher lean muscle mass as a proportion of body weight which diminished relative risk of prediabetes by 23% in non-T2D individuals [138]. Considering the etiology of T2D, muscle mass retention or accretion is implicit in controlling disease manifestation. In rats, muscle hypertrophy occurs alongside improvements in glucose disposal and 63% increase in glucose transporter protein-4 (GLUT-4) content [139]. Similarly, GLUT-4 content increases alongside gains in muscle mass and strength in T2D individuals [140]. Furthermore, in older adults with and without T2D, muscle accretion lowers myostatin (inhibitor of muscle accretion), partially restoring insulin sensitivity [141,142]. Given the role of adequate dietary protein as a key stimulus for muscle protein synthesis (MPS) and muscle mass retention/accretion, it is promising that longitudinal studies show inverse associations between higher protein intake and T2D development in middle-aged females [143]. Hence, adequate protein can support sustained intramuscular adaptation/remodeling for glucose handling and whole-body metabolic health.

Whilst dietary protein intake appears to be a pragmatic way to support and improve muscle mass and metabolic health in older individuals with T2D, it is important to note that protein requirements for these individuals may differ compared to younger individuals. Age-related blunted postprandial MPS (termed “anabolic resistance”) is exacerbated in obese older adults [144] but not necessarily by T2D per se [145]. Irrespective, T2D and obesity are often concomitant and worsened intramuscular insulin and anabolic signaling with aging means anabolic resistance among older adults with T2D is somewhat inevitable. For robust postprandial MPS stimulation that is critical for overall net muscle protein balance, it is generally accepted that older adults require higher protein doses than young adults [146,147,148,149] at a level that is greater than the current RDA of 0.8 g/kg/day [150]. Specifically, the dietary protein requirements for older individuals have been suggested to be at least 1.0–1.2 g/kg/day, and potentially higher in scenarios where physical activity is reduced [150,151]. The same is also true for older individuals with T2D, where protein intakes for optimal muscle anabolism may be even greater than for non-obese older adults due to a potentially aggravated muscle anabolic resistance [152].

#### 3.1.2. Dietary Protein-Induced Thermogenesis

Increasing dietary protein intake with the aim of supporting skeletal muscle retention in older individuals with T2D may facilitate weight management and healthy body composition with implications for thermogenesis. An increase in overall lean body mass increases metabolic rate due to loss of relatively dormant white adipose tissue and heightened energy cost associated with skeletal muscle metabolic processes [153,154,155]. Higher energy requirements are conducive to heat dissipation. Such loses are partially due to occasional inefficiencies in calcium release inhibition whereby, instead of moving myofibrils, energy is conserved as heat in non-shivering thermogenesis [156]. Heat can also be lost with diet-induced thermogenesis, particularly after protein ingestion [157]. Primarily however, skeletal muscle supports movements whereby greater muscle mass facilitates higher energy and heat dissipation [158]. Since positive energy balance precipitates weight gain, lowering energy balance through greater muscle mass and dietary protein consumption contributes to weight loss [159,160]. In turn, the reduction in body fat mass in older individuals with T2D could also, theoretically, alleviate postprandial muscle anabolic resistance and augment muscle retention over time, with implications for controlling T2D manifestation and comorbidities.

#### 3.1.3. Protein Ingestion and Insulinemia

Dietary protein ingestion can directly promote postprandial insulin secretion which may facilitate glucose regulation in older individuals with T2D. The insulinotropic effects of protein are mediated by the appearance of branched-chain amino acids (BCAAs; leucine, isoleucine, valine) which bind to pancreatic β-cell receptors. Indeed, providing low protein diets in healthy rats diminishes insulin secretion ~2-fold compared to high-protein diets [161]. Furthermore, this insulin concentration following high-protein diets is similar when low protein diets are supplemented with leucine, valine and isoleucine [161]. In humans, whey protein elicits a robust insulin response, the peak amplitude of which was reported to be ~50% and 25% greater than egg and fish ingestion, respectively [162] which can be attributed to the higher essential amino acid (EAA)/BCAA concentration of whey protein. In human trials, compared with placebo, ingesting 50 g of whey protein prior to a high-glycemic index breakfast elicited a 105% higher insulin secretion alongside a 28% reduction in post-prandial glycemia in older adults [163]. Despite the benefits, to minimize glucose excursions postprandially would require 50 g/meal of supplemental protein. Consuming such large protein doses with each meal could adversely impact appetite regulation and food intake at subsequent meals, the importance of which is discussed below. More recently, 15 g of intact whey protein preceding mixed-macronutrient meals at breakfast and lunch, containing 70% and 65% carbohydrate, respectively, significantly lowered interstitial glucose concentration compared to control in older males with T2D [164]. The glucose lowering effect of whey protein in this study was associated with an increased insulin response over a 3-h postprandial period.

Despite evidence of dietary protein intake lowering glycemia, numerous contrasting studies report lowered insulin sensitivity with high-protein diets in adults with excess adiposity [165,166] and T2D [106]. In fact, daily 7.5 g leucine supplementation over 6 months failed to improve glycemic control in older adults [167]. However, this may reflect that >7.5 g leucine is required to support improved glucose regulation. Some studies have reported a causal link between BCAA concentration and insulin resistance. Controlled studies have shown that lowering circulating BCAA concentrations lowers insulin concentration and improves lipolysis in middle-aged individuals with T2D [168], which could eventually restore metabolic flexibility [169]. Interestingly, in a separate cohort of middle-aged individuals with T2D, high-carbohydrate diets induced similar weight loss after 8 weeks compared to high-protein diets, yet significant improvements in insulin sensitivity were only seen with high-carbohydrate diets [170]. Conversely, increasing dietary protein to 30% of total energy intake, at the expense of carbohydrate and fat, elicits similar reductions in 10-h glucose area under the curve in individuals with T2D [171].

Although protein-induced insulinemia may acutely improve glucose handling, reports of impaired insulin sensitivity in the longer-term may be due to divergent BCAA profiles in protein sources. Recently, mouse models have identified that the BCAAs valine and isoleucine contribute to abnormal glucose handling [172]. However, all EAAs, especially leucine, are thought to be important as both signal and substrate for muscle anabolism. Henceforth, incorporating whole-food options which are high in leucine but lower in valine and isoleucine (e.g., egg, corn and wheat [173]) could benefit muscle anabolism and metabolic health outcomes, although this speculation remains to be confirmed in humans. Importantly, restricting certain protein sources is not conducive to muscle anabolism and retention or metabolic health improvements in older individuals with T2D. Alternative mechanisms to potentiate insulin secretion for glucose management relate to incretin peptides. Incretin peptides, such as glucagon-like peptide-1 (GLP-1) are secreted by the gut and bind to pancreatic cells to promote insulin secretion and inhibit glucagon secretion. Plant and animal proteins are associated with increased integrin response [174]. Henceforth, a combination of animal and plant proteins should be encouraged, as completely replacing plant for animal protein, or vice versa, would be unlikely to profoundly improve glucose handling [175,176].

#### 3.1.4. Appetite Regulation

Dietary protein is a highly satiating macronutrient. Appetite suppression with high-protein meals may effectively prevent or minimize calorie surplus and the excessive efflux of exogenous substrates which exacerbate T2D symptoms [177,178,179]. Feelings of satiety are derived from secretion of anorexigenic hormones, peptide YY and GLP-1. Controlled studies in humans have shown increases in peptide YY and GLP-1 negatively associate with energy intake and appetite [180,181,182]. Given its insulinotropic effects, GLP-1 inhibits acylated ghrelin (the ‘hunger hormone’), through insulinemia [183]. Although in normal weight humans, dietary fat elicits greater postprandial secretion of peptide YY and GLP-1 than carbohydrate [184,185], this difference is less apparent in obese populations [186]. Importantly, irrespective of body mass, dietary protein is a potent secretor of peptide YY and GLP-1 and can effectively lower energy intake [187]. Considering the impact of protein ingestion on anorexigenic hormone secretion, emphasizing dietary protein intake for obese individuals with T2D may have profound benefits for appetite regulation, weight management and glycemic control.

With isolated supplemental protein sources, divergent EAA profiles and digestibility may elicit distinct appetite responses [188]. For instance, slowly digested casein protein ingestion elicited higher GLP-1 secretion than whey protein over 6-h in obese adults [189]. However, sustaining satiety beyond 6-h is often not required, as shorter time frames typically separate main meals and mid-meal snacks. Henceforth, evidence showing that whey protein increases GLP-1 and suppresses appetite to a greater extent than casein over 3-h postprandial periods [190] makes this supplemental protein source an attractive option to maintain fullness when consumed between or with meals. Comparisons of whey to isolated plant proteins remain to be elucidated, although animal and cellular models highlight that wheat and pea protein increase GLP-1 acutely [191,192], although the temporal time-course and significance for glycemic regulation has not been translated to human models. Additionally, whey protein appears to augment satiety with whole food. Consuming a breakfast containing >50% protein from whole-foods increases satiety ~6-fold and anorexigenic hormones by 16–45% compared to lower protein breakfasts over 2-h postprandially [193]. The co-ingestion of 15 g whey protein with a mixed-meal breakfast in T2D warranted 17% higher satiety than co-ingesting with an isocaloric placebo [164]. Whilst supplemental protein is the most feasible way to elevate total protein intake, a ‘food first’ approach should be incorporated when combatting metabolic disease. Dietary proteins are effective in prolonging fullness in obese populations [194], likely due to bioactive peptides within protein-rich food, which further augment satiety [195]. Henceforth, elevating protein intake through dietary and supplemental sources will lessen appetite response and promote fat loss through lowered energy contribution from other macronutrients in order to improve indices of T2D progression [196,197].

### 3.2. Branched-Chain Amino Acids

BCAAs make up approximately 40% of circulating amino acids and play an important role in protein turnover and, as previously discussed, have an insulinotropic effect [198,199,200]. Due to the complications of T2D, individuals are at risk of developing sarcopenia [201]. Decreased muscle mass inhibits glucose uptake, metabolic functioning, and individual’s physical capacity which may exacerbate the effects of T2D and sarcopenia. For this reason, supplementation with BCAAs may benefit individuals with T2D by stimulating insulin secretion and protein synthesis, inevitably regulating blood glucose disposal and maintaining/increasing muscle mass and function [202].

Ingestion of BCAAs increases insulin secretion from pancreatic β-cells, potentially through the incretin effect [203,204]. It is hypothesized that BCAAs stimulate the secretion of peptide hormones glucagon-like peptide 1 (GLP-1) and glucose dependent insulinotropic polypeptide (GIP) [203]. GLP-1 and GIP work together to simulate insulin secretion postprandially, improving glycemic control and β cell growth while decreasing β cell apoptosis [205]. Function of GLP-1 and GIP is compromised in T2D, but research suggests BCAA ingestion stimulates inhibited GLP-1 and GIP for improved insulin secretion [203,204,206]. Special consideration has been given to leucine and its metabolites due to its insulinotropic effects and stimulation of the mammalian target of rapamycin (mTOR) pathway which regulates protein synthesis, cell growth, and fuel metabolism [203,207,208,209]. Specifically, rapamycin-sensitive (mTORC1), a component of mTOR, is activated in response to an increase in cellular levels of leucine [209,210]. This signaling pathway elicits mRNA translation and protein synthesis while decreasing muscle wasting and cell death when activated [208,211]. Inhibition of mTOR signaling can occur through the activation of AMP-activated protein kinase or by upstream phosphate and tension homology. This can decrease insulin secretion, beta-cell activation and lead to the development of proinflammatory cytokines (IL-6, IL-Iβ, TNF-α) [212]. Long term leucine supplementation can also increase insulin secretion through improved gene expression and metabolic function [213]. BCAA infusion has been shown to activate p70S6 kinase and 4E-BP, downstream protein kinases in the mTOR pathway, leading to an increased capacity for muscle protein synthesis [214,215]. Increased protein synthesis is greatly beneficial for individuals with T2D, as skeletal muscle can account for up to 80% of glucose uptake, which will aid in regulation of blood glucose levels and combats muscle wasting that occurs due to complications of the disease and aging [202].

### 3.3. Creatine

Supplementing with creatine, a nitrogen-containing organic acid, has been shown to alter body composition [216,217,218,219,220] and may potentially enhance glucose control and decrease insulin resistance [221]. Creatine is endogenously synthesized from reactions involving the amino acids arginine, glycine, and methionine in the kidneys and liver [222]. Creatine can also be consumed in the diet (1–2 g/day) primarily from red meat, seafood, and poultry [222,223,224] or through dietary supplements. Once creatine enters circulation, it is transported to various tissues, with the majority (~95%) taken up (by way of the SLC6A8 transporter) and stored in skeletal muscle, and the remaining (~5%) found in other tissues, such as cardiac myocytes, retina, neurons, and testicles. In the muscle, ~2/3rd is converted to phosphocreatine (PCr) and 1/3rd remains as free creatine [222]. Following repeated muscle contractions (i.e., exercise), adenosine diphosphate (ADP) is rapidly phosphorylated by PCr to resynthesize adenosine triphosphate (ATP) in order to maintain energy status [225]. Creatine supplementation elevates intramuscular stores ~20% more than habitual dietary practices [226,227], which in turn may increase PCr resynthesis and recovery during exercise. Creatine may influence muscle biology by stimulating cellular hydration status, calcium kinetics, glycogen content, muscle protein kinetics, satellite cells, growth factors, inflammation and oxidative stress, which have been implicated in enhancing muscle health (for reviews see [216,220,225,228]. Creatine also plays a role in thermogenesis and whole body energy expenditure [229,230,231], which is associated with adiposity.

In healthy older adults, supplementing with creatine during a resistance training program increased muscle mass (+1.37 kg) and decreased fat mass (0.5 kg) more than placebo and resistance training [218,229]. Individuals with T2D often have low muscle quantity and increased adiposity; therefore it appears that creatine combined with exercise may be a viable strategy to enhance body composition. However, research examining the efficacy of creatine and exercise individuals with T2D is limited. In female Wistar rats, creatine supplementation for 3 weeks increased glycogen content, GLUT-4 mRNA, AMPK phosphorylation, and GLUT-4 content [232]. However, in humans, van Loon et al. [233] found no effect of creatine (20 g/day for 5 days followed by 2 g/day for 37 days) on GLUT-4 mRNA or protein content. In an open-label, crossover design (with a short 2-day washout period) in a small sample of T2D patients, creatine (2 g provided twice a day for 5 days) had a similar glucose lowering effect as metformin [234]. Only two studies have assessed the effects of creatine in conjunction with exercise in T2D patients [235,236,237]. Gualano et al. [236] showed that the combination of creatine (5 g/day for 12 weeks) and exercise (aerobic and resistance training, 3x/week) resulted in significant reductions in HbA1c and glycaemia during a meal tolerance test and increased GLUT-4 translocation compared to placebo in in older individuals (>50 yrs) with T2D. Using the same creatine and exercise intervention, Alves et al. [235] found an increase in AMPK (*p* = 0.06) which was associated with changes in GLUT-4 translocation and Hb1Ac levels in older individuals with T2D.

Collectively, creatine supplementation and exercise have the potential to provide beneficial effects for older individuals with T2D.

### 3.4. Vitamin D

Vitamin D is a fat-soluble hormone that plays an important role in the regulation of circulating calcium and phosphates, gene transcription, inflammatory response, and glucose metabolism [238,239]. Vitamin D is endogenously produced from exposure to sunlight or obtained through dietary and/or supplementation practices [240,241,242]. Exogenous sources (diet or supplementation) of vitamin D exist in two forms, ergocalciferol (Vitamin D_2_) and cholecalciferol (Vitamin D_3_), whereas endogenous synthesis of vitamin D is referred to as vitamin D_3_ [242,243]. Briefly, once in circulation, vitamin D binds with vitamin D-binding protein (DBP) and is transformed by D25-hydroxylase in the liver to 25-hyroxyvitamin D (25(OH)D) [241]. Researchers measure this form of circulating vitamin D as an indicator of both sun exposure and dietary vitamin D levels within the body [238,239,243]. Circulating 25(OH)D is converted in the kidneys, through regulation of the parathyroid hormone, by 25-hydroxyvitamin D-1alpha-hydroxylase (CYP27B1) enzyme to its active form 1,25-dihydroxyvitamin D (1,25(OH)_2_D) [241]. 1,25(OH)_2_D binds to vitamin D receptor (VDR) which can be found in several cells including the liver, adipose tissue, skeletal muscle, and pancreatic beta cells [244].

The active form of vitamin D can regulate the inflammatory response by inhibiting production of pro-inflammatory cytokines (tumor necrosis factor-α, interleukin-1β and interleukin-6) and upregulates anti-inflammatory cytokines (interleukin-6) [245,246]. Inflammation in conjunction with prolonged hyperglycemia leads to over production of reactive oxygen species within the body, causing oxidative stress and microvascular and macrovascular complications [247]. In individuals with T2D, chronic low-grade inflammation is found in the liver, skeletal muscle, adipose tissue and pancreas which can cause tissue damage, further impairing the body’s ability to regulate insulin levels [248].

Vitamin D deficiency can result from decreased utilization of available vitamin D in the body, inadequate dietary intake, decreased efficiency of cutaneous synthesis with age, geographic latitude, and/or limited sun exposure due to season, time of the day, and weather conditions [243]. Low levels of circulating vitamin D have been shown to be inversely related to BMI, and it is suggested the majority of vitamin D, as a fat-soluble hormone, may become sequestered in adipose tissue decreasing circulating levels [249]. An inverse relationship has also been indicated between vitamin D deficiency and insulin resistance and impaired beta cell function [250,251]. Vitamin D deficiency is prevalent in individuals with T2D and may pay a key role in the onset of the disease by impacting insulin secretion, fasting glucose levels, and systemic inflammation [252,253,254]. Deficiency in individuals with T2D is associated with impaired glucose homeostasis and both microvascular and macrovascular complications [255]. Observational studies have found a heightened risk for incidents of diabetes, of up to 50%, for individuals in the lowest category of vitamin D serum levels versus the highest [256,257].

Currently a consensus on the optimal vitamin D concentration or supplementation for deficiency does not exist [258,259]. The Scandinavian Nutrition Societies, the European Society for clinical and Economic Aspect of Osteoporosis and Osteoarthritis, the North American Institute of Medicine, the German Osteology governing body (DVO) and D-A-CH nutrition societies have agreed that a circulating level of 25(OH)D should not be lower than 50 nmol/L, and below 25–30 nmol/L indicates deficiency [246,260,261].

**Figure 2:**
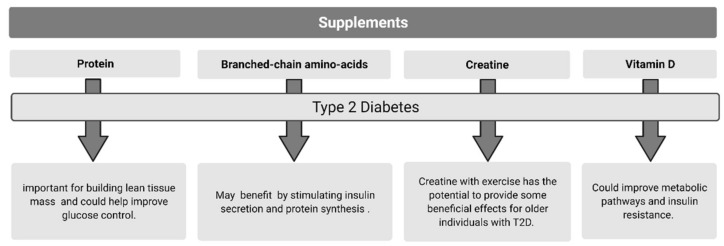
Purported supplements to improve type 2 diabetes.

Studies investigating the efficacy of vitamin D supplementation have demonstrated significant effects on circulating vitamin D, total cholesterol, glycated hemoglobin (HbA1c), low density lipoproteins (LDL), homeostatic model assessment (HOMA-IR), and triglyceride levels, all leading to improving metabolic pathways and insulin resistance [246,262]. It is suggested that vitamin D supplementation in individuals with T2D may need to be significantly higher to achieve an adequate 25(OH)D levels due to the increased amount of adipose tissue and lower base vitamin D levels [258,263]. A review conducted by Li et al. [258] found that most studies in individuals with T2D used vitamin D at 2000 IU/day, which elicited improvements in glycemic control. A meta-analysis review by Mirhosseini et al. [262] suggested a minimal dose of 4000 IU/day was required to elicit positive effects on HbA1c, HOMA-IR, fasting plasma glucose, and to reach an adequate level of circulating 25(OH)D. Additional systematic reviews report improvements in insulin resistance and fasting plasma glucose following vitamin D supplementation in individuals with T2D [253,264,265]; whereas Morieira-Lucas et al. [266] found a significant increase in 25(OH)D but no changes in beta cell function, HbA1c, or fasting insulin following 24 weeks of 4000 IU of vitamin D supplementation. These results mirror a study conducted by Wagner et al. [267] showing no change in insulin sensitivity or glucose tolerance from 30,000 IU/week of vitamin D supplementation. The difference in results may be attributed to due to study duration, sample size, and various dosage levels of vitamin d [253,264,265]. It is also important to note that excessive ingestion of vitamin D can be toxic and cause hypercalcemia and hypercalciuria [268,269]. Figure 2 summarizes dietary strategies used to improve metabolic outcomes in Type 2 Diabetes.

## 4. Conclusions

Overall, there appears to be some overlapping beneficial effects from various dietary and supplementation interventions in individuals with T2D. The MedDiet improves metabolic health and is suitable in the management of T2D. A plant-based diet shows promise in the prevention and management of diabetes and its complications but still requires further investigation. Over the short-term, caloric restriction improves glucose control and beta cell function in individuals with T2D, with the possibility of T2D remission seen with very low-calorie diets. The impacts of IF on glycemic control remain less clear. Low-carbohydrate and very low-carbohydrate diets can improve glycemic control and, at least in the shorter term, result in weight loss likely due to reduced calorie intake. A low-GI diet appears to help manage glucose levels and parameters associated with T2D, but is most likely to be due to the inclusion of well-balanced, minimally processed and high fiber foods. Furthermore, protein intake greater than the RDA is important for building lean tissue mass (main tissue of glucose disposal) and could help improve glucose control through multiple mechanisms. Lastly, BCAA, creatine, and vitamin D appear to show promise for individuals with T2D, but further research, especially long-term RCTs, is needed before consensus on the efficacy of these select supplements for individuals with T2D can be arrived at.

## Figures and Tables

**Table 1:** Definition of terms used to describe different types of fasting or calorie restricted eating patterns (adapted from [76]).

Eating Patterns	Definition
Intermittent Fasting	Involves fasting for varying periods of time, typically for at least 12 h or longer.
Time Restricted Eating	Involves restricting food intake to specific time periods of the day, typically between an 8–12 h period each day (e.g., 16:8, involving 16 h fasting and an 8 h eating window)
Alternate Day Fasting	Involves consuming no calories on fasting days and alternating fasting days with a day of unrestricted food intake or “feast” day
Alternate Day Modified Fasting	Involves consuming less than 25% of baseline energy needs on “fasting” days, alternate with a day of unrestricted food intake or “feast” day
Periodic Fasting	Consists of fasting only 1 or 2 days a week and consuming food ad libitum on 5 to 6 days per week.
Fasting Mimicking Diet	Consists of a very low calorie (usually high fat) diet designed to replicate a fasting state, often done periodically or in cycles.
